# Oxidized Low-Density Lipoprotein and Its Role in Immunometabolism

**DOI:** 10.3390/ijms252111386

**Published:** 2024-10-23

**Authors:** Negin Mosalmanzadeh, Brandt D. Pence

**Affiliations:** 1 College of Health Sciences, University of Memphis, Memphis, TN 38111, USA; nmslmnzd@memphis.edu; 2 College of Health Sciences and Center for Nutraceutical and Dietary Supplement Research, University of Memphis, Memphis, TN 38111, USA

**Keywords:** OxLDL, immunology, metabolism, inflammation, senescence, atherosclerosis, aging

## Abstract

Modified cholesterols such as oxidized low-density lipoprotein (OxLDL) contribute to atherosclerosis and other disorders through the promotion of foam cell formation and inflammation. In recent years, it has become evident that immune cell responses to inflammatory molecules such as OxLDLs depend on cellular metabolic functions. This review examines the known effects of OxLDL on immunometabolism and immune cell responses in atherosclerosis and several other diseases. We additionally provide context on the relationship between OxLDL and aging/senescence and identify gaps in the literature and our current understanding in these areas.

## 1. Introduction

Atherosclerosis is a pathophysiological condition characterized by the gradual thickening of the arterial intima, leading to decreased elasticity, narrowing, and reduced blood supply. This condition affects the arterial walls, resulting in clinical manifestations such as angina pectoris, myocardial infarction, and cerebral infarction [1]. One of the hallmark features of atherosclerotic lesions is the presence of lipid-laden foam cells [2]. These foam cells arise from the oxidation of low-density lipoprotein (LDL), which triggers the generation of various oxidative byproducts that play crucial roles in the early stages of atherosclerosis. This occurs through the recruitment of monocyte-derived macrophages into the arterial wall and the promotion of intracellular cholesterol ester accumulation within these cells, resulting in foam cell formation [3].

Low-density lipoprotein (LDL) is a key type of lipoprotein that plays a central role in lipid metabolism. Its primary function is to transport cholesterol from the liver to various tissues and organs throughout the body, where cholesterol is used for the maintenance of cell membranes and the production of certain hormones [4,5]. LDL is essential for normal cellular function, but when present in excessive amounts, it can accumulate in the arterial walls, leading to the development of cardiovascular diseases [6].

Oxidized low-density lipoprotein (OxLDL) is a modified form of LDL that has undergone oxidative changes, significantly altering its biochemical properties and physiological functions [7]. However, when LDL undergoes oxidation, it contributes to the development of atherosclerosis, a leading cause of cardiovascular diseases [8,9]. OxLDL is not merely a marker of oxidative stress but also actively participates in inflammatory processes by interacting with various immune cells [10].

The significance of OxLDL extends to autoimmune diseases such as antiphospholipid syndrome (APS), in which it forms complexes with β2-glycoprotein I (β2GPI), recognized by antiphospholipid antibodies (aPL) [11,12]. These complexes are involved in arterial and venous thromboembolic complications and pregnancy morbidity associated with APS [13]. The presence of OxLDL/β2GPI complexes is also noted in other systemic autoimmune diseases, such as systemic lupus erythematosus (SLE) and systemic sclerosis (SSc) [14].

Immunometabolism is an emerging field that explores the intersection between immune cell function and metabolic processes. This interplay is crucial for understanding health and disease because metabolic reprogramming within immune cells influences their differentiation, activation, and function, impacting overall immune responses [15]. Immunometabolism provides a framework to understand how metabolic alterations can drive immune responses and contribute to chronic inflammatory diseases.

The rationale for linking OxLDL with immunometabolism lies in their shared roles in promoting inflammation and metabolic dysregulation [16]. Understanding how OxLDL influences immunometabolism can reveal novel insights into the pathogenesis of metabolic and cardiovascular diseases. This connection is particularly relevant for conditions such as atherosclerosis and autoimmune diseases, where OxLDL and immune cell interactions play pivotal roles in disease progression [17,18].

The aim of this review is to examine the role of OxLDL in immunometabolism. This includes exploring its biochemical and physiological properties and interactions with immune cells and discussing the clinical and therapeutic implications of these interactions. Additionally, this review will highlight future research directions to address current gaps in knowledge.

## 2. Methods of LDL Oxidation

LDL oxidation occurs through both enzymatic and non-enzymatic pathways. These pathways are crucial in the progression of atherosclerosis, and the enzymes involved play distinct roles in various metabolic and inflammatory processes [19].

Enzymatic oxidation of LDL involves key enzymes that are generated in different immune and metabolic pathways. Lipoxygenases are produced primarily in immune cells, such as leukocytes and macrophages, and are involved in the metabolism of arachidonic acid, leading to the formation of inflammatory mediators like leukotrienes [20]. Lipoxygenases play a significant role in inflammation and immune responses, particularly in the context of immunometabolism, where they contribute to the inflammatory processes involved in LDL oxidation [20,21]. Myeloperoxidase is produced by neutrophils and macrophages and is involved in the production of hypochlorous acid (HOCl), a potent oxidant [22,23]. Myeloperoxidase is crucial for the immune response to infections and is a key contributor to oxidative stress and inflammation, which are central to the oxidation of LDL [24]. NADPH oxidase is an enzyme complex found in immune cells like macrophages and neutrophils, where it plays a vital role in generating reactive oxygen species (ROS) during the respiratory burst [25]. The production of ROS by NADPH oxidase is critical for inflammation and pathogen defense, and it significantly contributes to LDL oxidation. This enzyme is integral to the processes of inflammation and oxidative stress, which drive atherosclerosis [26]. The enzymatic oxidation pathways, particularly through immune and inflammatory responses, underscore the critical role of these processes in immunometabolism, as well as in the broader context of cardiovascular diseases.

Non-enzymatic oxidation of LDL occurs through autoxidation processes, often in the presence of metal ions like copper and iron [27]. These metal ions catalyze the formation of reactive oxygen species (ROS), which initiate the oxidation of lipids and proteins in LDL [28,29]. The non-enzymatic oxidation of LDL tends to occur in environments with high oxidative stress, such as within the subendothelial space of arteries. Here, elevated levels of ROS and metal ions create a pro-oxidative environment that promotes the modification of LDL into oxidized LDL. Non-enzymatic oxidation is also influenced by factors such as diet, smoking, and the presence of systemic oxidative stress [27,30].

Both enzymatic and non-enzymatic pathways contribute to the formation of OxLDL, which has pro-inflammatory and pro-atherogenic properties. This drives the formation of foam cells and promotes the development of atherosclerosis.

## 3. Biochemical and Physiological Properties of OxLDL

LDL is the major lipid carrier in plasma, consisting of cholesteryl esters, phospholipids, free cholesterol, triglycerides, and apolipoprotein B100 (apoB100) [4]. ApoB100 is a large, highly insoluble protein synthesized in the liver [31]. Under normal physiological conditions, LDL transports cholesterol to peripheral tissues. However, when LDL is exposed to reactive oxygen species (ROS) and other oxidative agents, particularly within the subendothelial space of arterial walls, it undergoes oxidative modifications [32]. This oxidative modification leads to the formation of OxLDL [33,34], which is characterized by fragmented apoB, oxidized lipids, and various aldehyde and ketone derivatives [35].

**Figure 1:**
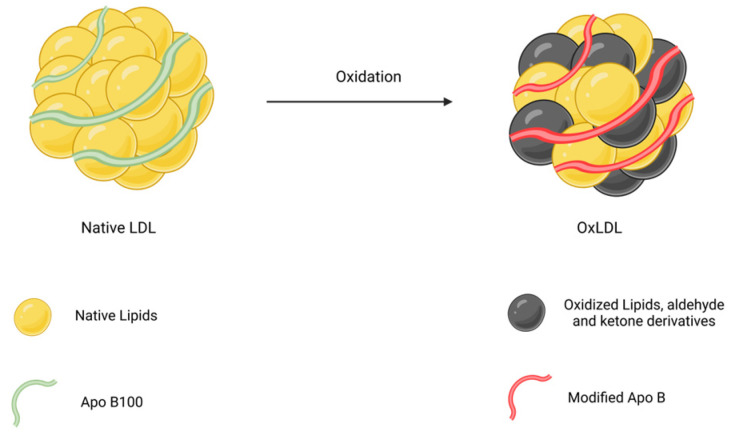
Schematic Representation of LDL Oxidation. Created with biorender.com.

The formation of OxLDL involves interactions with ROS and oxidative enzymes, resulting in structural and functional changes [36]. Key features of OxLDL include the oxidation of phospholipids, cholesterol esters, and apoB100, leading to the creation of lipid peroxides, malondialdehyde (MDA), and other reactive aldehydes that can further alter proteins and lipids within LDL [37]. The heterogeneous nature of OxLDL, with varying proportions of oxidized lipids, complicates the understanding of its overall toxic effects [38]. A basic schematic of LDL oxidation is shown in Figure 1.

The concept that LDL must undergo structural changes to become atherogenic was initially proposed by Brown and Goldstein in 1983 [39]. They discovered that macrophages could uptake modified LDL even in the absence of native LDL receptors, leading to cholesterol accumulation and foam cell formation. Goldstein et al. identified the acetyl LDL receptor responsible for modified LDL uptake [40], later cloned as scavenger receptor A (SR-A) by Kodama et al. in 1988 [41]. Other scavenger receptors and LDL modifications, such as oxidation and complexing with immunoglobulins, have since been identified [42,43,44,45].

The susceptibility of LDL to oxidation depends on its composition, including the presence of polyunsaturated fatty acids (PUFA), preformed peroxides, and antioxidants [46]. An LDL particle’s core typically contains cholesteryl esters, triglycerides, and a few antioxidants, while its outer layer comprises cholesterol, phospholipids, and tocopherol. Variations in diet and pathological conditions influence the PUFA content and oxidation susceptibility of LDL [47].

During the initial phase of LDL oxidation, oxidants first interact with the most oxidizable compounds, such as antioxidants and PUFA [48]. Depending on their relative concentrations, antioxidants may either delay or promote PUFA oxidation [49]. The initiation of PUFA peroxidation produces lipid radicals and conjugated dienes, which can react with oxygen to form peroxyl radicals and other derivatives, propagating lipid peroxidation and leading to extensive lipid oxidation [50]. The oxidation reaction concludes with the formation of stable lipid peroxidation products and intermolecular links.

Aldehydes produced during lipid peroxidation can react with apoB, progressively modifying it and altering its receptor interactions. Minimally or mildly oxidized LDL has low levels of lipid peroxidation products and minor apoB alterations, allowing for its uptake through the apoB/E receptor [51]. In contrast, extensively oxidized LDL exhibits significant lipid oxidation and apoB modifications, reducing its affinity for the apoB/E receptor and increasing it for scavenger receptors [35,52].

Atherosclerotic lesions contain OxLDL with reduced PUFA content, oxidized PUFA, lipid peroxidation products like aldehydes and oxysterols, and modifications of apoB by oxidized lipids. These modifications contribute to the development and progression of atherosclerosis.

## 4. Overview of Immunometabolism

Immunometabolism encompasses the study of how metabolic processes influence immune cell function and how immune responses, in turn, affect metabolic pathways [53]. Immune cells rely on distinct metabolic programs to support their activation, proliferation, and effector functions. These metabolic programs include glycolysis, oxidative phosphorylation, fatty acid oxidation, and amino acid metabolism [54].

Glycolysis, the process of breaking down glucose to produce energy, is rapidly upregulated in activated immune cells. This metabolic shift, known as the Warburg effect, supports the biosynthetic and energetic demands of proliferating cells [55,56]. Aerobic glycolysis is particularly prominent in activated T cells, macrophages, and dendritic cells. In contrast, quiescent and regulatory immune cells rely more on oxidative phosphorylation and fatty acid oxidation to meet their energy needs [57,58].

Metabolic pathways also play a crucial role in immune cell differentiation. For example, the differentiation of pro-inflammatory M1 macrophages is associated with increased glycolysis and reduced mitochondrial oxidative phosphorylation [59,60]. Conversely, anti-inflammatory M2 macrophages depend on oxidative phosphorylation and fatty acid oxidation [60,61]. Similar metabolic reprogramming occurs in T cells, where effector T cells (Teff) rely on glycolysis, while regulatory T cells (Treg) utilize oxidative phosphorylation [62,63,64].

By understanding the principles of immunometabolism, researchers can identify potential therapeutic targets to modulate immune cell metabolism and treat inflammatory diseases. This approach offers the potential for novel treatments that address the metabolic underpinnings of immune responses, thereby improving outcomes in conditions such as atherosclerosis and diabetes.

## 5. Interaction of OxLDL with Immune Cells

OxLDL is recognized by immune cells through various receptors, leading to diverse functional outcomes. Macrophages and dendritic cells are particularly responsive to OxLDL due to their expression of scavenger receptors. The binding of OxLDL to these receptors initiates a cascade of cellular events that influence immune cell function and phenotype [65,66].

Scavenger receptors are a class of receptors expressed on the surface of various immune cells, including macrophages and dendritic cells. These receptors are primarily responsible for the recognition and internalization of modified forms of LDL, including OxLDL. Key scavenger receptors involved in OxLDL uptake include CD36, SR-A, and LOX-1 [65,67]. CD36 is a class B scavenger receptor widely expressed in macrophages, dendritic cells, and other cell types. It binds to oxidized phospholipids and oxidized cholesterol esters present in OxLDL, facilitating its uptake and triggering pro-inflammatory signaling pathways, contributing to foam cell formation and inflammation [68]. SR-A, a class A scavenger receptor expressed in macrophages, includes different types such as SR-AI and SR-AII, both involved in binding and internalizing OxLDL. SR-A-mediated uptake leads to the accumulation of lipid droplets within macrophages, promoting foam cell formation and the development of atherosclerotic plaques [69]. LOX-1 is a type II membrane protein expressed on endothelial cells, macrophages, and smooth muscle cells. It specifically binds to OxLDL and mediates its internalization, facilitating lipid uptake and activating oxidative stress and inflammatory responses [70,71]. SR-BI, another class B scavenger receptor, is involved in the selective uptake of cholesteryl esters from high-density lipoproteins (HDL) but also binds OxLDL, contributing to lipid accumulation in macrophages [72,73].

In macrophages, OxLDL uptake via scavenger receptors results in foam cell formation. The internalization of OxLDL triggers signaling pathways that promote lipid accumulation, oxidative stress, and the secretion of pro-inflammatory cytokines [69,74]. Once internalized, OxLDL is transported to the lysosomes, where enzymes such as acid lipase degrade its lipid components, including cholesteryl esters, into free cholesterol and fatty acids. Within the lysosome, cholesteryl esters undergo hydrolysis, releasing free cholesterol [69]. Box 1 summarizes foam cell biology.

Box 1 **Foam cells.**

Foam cells are lipid-laden macrophages or smooth muscle cells that result from the uptake of OxLDL. These cells play a pivotal role in the initiation and progression of atherosclerosis, a leading cause of cardiovascular diseases. The accumulation of foam cells in the arterial walls forms the core of atherosclerotic plaques.

 

Formation and Role:

Foam cells form when macrophages internalize OxLDL via scavenger receptors such as CD36 and SR-A. Once inside the macrophages, OxLDL is degraded, leading to the accumulation of cholesterol esters and lipids, giving the cell a foamy appearance. These cells are central to the development of fatty streaks, the earliest visible sign of atherosclerosis.

 

Impact on Inflammation:

Foam cells secrete inflammatory cytokines and chemokines that recruit more immune cells, perpetuating the inflammatory cycle and contributing to the growth and instability of atherosclerotic plaques. This continuous inflammation leads to plaque rupture, potentially causing heart attacks or strokes.

 

Clinical Significance:

Foam cells are key drivers in atherosclerosis, making them crucial targets for therapeutic intervention. Strategies aimed at reducing foam cell formation, such as inhibiting OxLDL uptake or enhancing cholesterol efflux, are potential approaches to prevent plaque formation and progression.

The free cholesterol is then transported out of the lysosome into the cytoplasm, where it can either be used for membrane synthesis or subjected to re-esterification by the enzyme acyl-CoA: cholesterol acyltransferase (ACAT) [69,75]. This enzyme converts free cholesterol into cholesteryl esters for storage within lipid droplets. The accumulation of these cholesteryl esters in lipid droplets contributes to the transformation of macrophages into foam cells. The uptake of OxLDL generates ROS within macrophages, inducing oxidative stress that can damage cellular components, including proteins, lipids, and DNA, and activate redox-sensitive signaling pathways such as NF-κB, promoting the expression of pro-inflammatory genes [76,77]. Foam cells secrete pro-inflammatory cytokines, including TNF-α, IL-1β, and IL-6, which contribute to the inflammatory milieu within atherosclerotic plaques and recruit additional immune cells to the site, perpetuating inflammation and plaque growth [77].

Dendritic cells also internalize OxLDL through scavenger receptors, affecting their antigen-presenting capacity and cytokine production [65,78]. OxLDL uptake by dendritic cells can enhance their maturation and activation, leading to increased presentation of oxidized lipid antigens to T cells. This interaction promotes the differentiation of pro-inflammatory T cells, further amplifying the immune response [65,78]. Dendritic cells process internalized OxLDL and present oxidized lipid antigens on major histocompatibility complex (MHC) molecules [79,80], crucial for the activation of T cells, particularly CD4+ T cells that can differentiate into various subsets, including Th1 and Th17 cells, known for their pro-inflammatory roles. The interaction of OxLDL with dendritic cells leads to the production of cytokines such as IL-12 and IL-23, important for the differentiation and maintenance of Th1 and Th17 cells, respectively. These T cell subsets produce pro-inflammatory cytokines like IFN-γ and IL-17, contributing to chronic inflammation in atherosclerosis [80,81].

OxLDL modulates the phenotype and function of immune cells through various mechanisms. The uptake of OxLDL induces oxidative stress by generating ROS, which can damage cellular components and activate redox-sensitive signaling pathways, promoting the inflammatory phenotype of immune cells [82,83]. Additionally, OxLDL disrupts mitochondrial function, leading to the release of pro-apoptotic factors, like cytochrome c, which activate caspases and trigger programmed cell death (apoptosis) [84]. OxLDL also induces endoplasmic reticulum (ER) stress, activating the unfolded protein response (UPR); excessive ER stress can lead to apoptosis. OxLDL alters metabolic pathways within immune cells, promoting glycolysis and lipid metabolism to support the energetic and biosynthetic demands of activated immune cells, enhancing their inflammatory potential [85,86]. Additionally, OxLDL can activate the NLRP3 inflammasome in macrophages, leading to the production of IL-1β and IL-18, cytokines that play key roles in promoting inflammation and have been implicated in the pathogenesis of atherosclerosis [87,88,89]. Furthermore, OxLDL disrupts cholesterol homeostasis by interfering with mechanisms that regulate intracellular cholesterol levels, such as efflux to HDL via ATP-binding cassette transporters like ABCA1 and ABCG1. This disruption leads to cholesterol and ester accumulation, contributing to foam cell formation [90]. OxLDL triggers the expression of adhesion molecules such as VCAM-1 and ICAM-1 on endothelial cells, mediating the adhesion and transmigration of leukocytes into the arterial intima, promoting the inflammatory response and plaque formation [91,92,93].

The recognition and internalization of OxLDL by immune cells have significant implications for disease pathogenesis [83,91]. By driving foam cell formation, oxidative stress, and pro-inflammatory cytokine production, OxLDL contributes to the development and progression of atherosclerosis [83,94]. Understanding these interactions provides insights into the molecular mechanisms underlying chronic inflammatory diseases and identifies potential targets for therapeutic intervention. The continuous recruitment of immune cells and the accumulation of foam cells contribute to plaque growth. The inflammatory environment within the plaque can weaken its fibrous cap, making it more prone to rupture, which can lead to thrombosis and acute cardiovascular events [95,96,97]. The cytokines and chemokines produced by OxLDL-stimulated immune cells can have systemic effects, contributing to chronic low-grade inflammation associated with metabolic syndrome, diabetes, and other chronic conditions [96,98].

**Figure 2:**
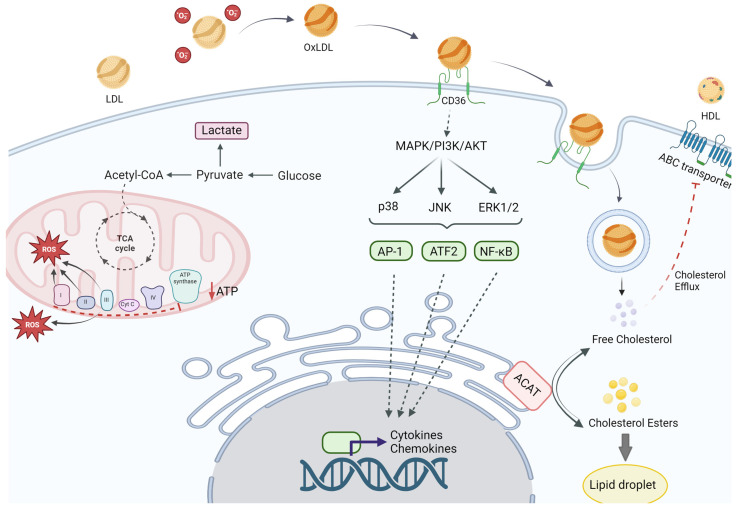
Signaling mechanisms related to OxLDL recognition in myeloid cells. Created with biorender.com.

A basic schematic of cellular recognition of OxLDL and resulting signaling pathways is shown in Figure 2.

## 6. Mechanistic Insights into OxLDL and Immunometabolic Pathways

### 6.1. Metabolic Reprogramming Triggered by OxLDL

OxLDL induces metabolic reprogramming in immune cells, promoting metabolic flexibility to adapt to oxidative stress and inflammatory conditions [99]. This reprogramming involves shifts in energy production pathways, such as increased glycolysis and altered lipid metabolism [85,99]. The ability of immune cells to switch between different metabolic states enables them to respond effectively to environmental changes and maintain their functions [85,100].

### 6.2. Influence on Aerobic Glycolysis

OxLDL significantly impacts the metabolic processes within immune cells, particularly influencing aerobic glycolysis. Aerobic glycolysis, also known as the Warburg effect, is a metabolic shift where cells preferentially utilize glycolysis over oxidative phosphorylation for ATP production, even in the presence of ample oxygen [85,99]. This shift supports the biosynthetic and energetic demands of rapidly proliferating cells, such as activated immune cells.

When immune cells, such as macrophages and dendritic cells, internalize OxLDL, it triggers several signaling pathways that enhance glycolytic activity. This process involves the upregulation of key glycolytic enzymes and transporters, facilitating increased glucose uptake and metabolism [101]. Key enzymes involved in this process include hexokinase, phosphofructokinase (PFK), and pyruvate kinase, which catalyze critical steps in the glycolytic pathway [102,103]. Hexokinase catalyzes the phosphorylation of glucose to glucose-6-phosphate, the first step in glycolysis. OxLDL upregulates hexokinase expression, increasing glycolytic flux [57,104]. PFK is a rate-limiting enzyme in glycolysis that converts fructose-6-phosphate into fructose-1,6-bisphosphate. OxLDL enhances PFK activity, promoting glycolysis [102,104,105]. Pyruvate kinase catalyzes the final step in glycolysis, converting phosphoenolpyruvate to pyruvate, generating ATP. OxLDL-induced signaling pathways upregulate pyruvate kinase and facilitating rapid ATP production [57,102,104].

One of the key regulators of this metabolic shift is hypoxia-inducible factor-1α (HIF-1α), a transcription factor that promotes the expression of glycolytic enzymes and glucose transporters. OxLDL can stabilize HIF-1α even under normoxic conditions, mimicking a hypoxic response. Stabilized HIF-1α translocates to the nucleus and induces the expression of genes involved in glycolysis, enhancing the glycolytic capacity of immune cells [106,107].

The increased glycolytic flux driven by OxLDL provides rapid ATP production and generates metabolic intermediates required for various biosynthetic processes, such as nucleotide and amino acid synthesis, essential for cell proliferation and function. This metabolic reprogramming supports the energetic demands of immune activation and the production of pro-inflammatory cytokines. In macrophages, OxLDL-induced glycolysis leads to the production of pro-inflammatory cytokines and perpetuates the chronic inflammatory state [57,99]. In dendritic cells, OxLDL-enhanced glycolysis supports their maturation, facilitating the presentation of antigens to T cells, promoting the differentiation of pro-inflammatory T cell subsets and thus amplifying the immune response [57,101].

### 6.3. Impact on Fatty Acid Metabolism

OxLDL has profound effects on fatty acid metabolism within immune cells, significantly altering lipid accumulation and modifying lipid signaling pathways. The internalization of OxLDL by immune cells, such as macrophages and dendritic cells, leads to the accumulation of oxidized lipids. These lipids can disrupt normal cellular signaling and function, thereby influencing immune cell differentiation and activation and contributing to the inflammatory response [68,86].

When OxLDL is internalized by immune cells via scavenger receptors, the lipids within OxLDL are processed and stored within the cells. This internalization results in the accumulation of oxidized lipids, particularly cholesteryl esters and triglycerides, within lipid droplets in the cytoplasm. The enzyme acyl-CoA:cholesterol acyltransferase (ACAT) plays a crucial role in re-esterifying free cholesterol into cholesteryl esters, promoting their storage in lipid droplets. This accumulation of lipids within immune cells, particularly macrophages, leads to the formation of foam cells [68,108].

The presence of oxidized lipids within immune cells affects various lipid signaling pathways. Oxidized fatty acids and other lipid oxidation products can act as ligands for peroxisome proliferator-activated receptors (PPARs), which are nuclear receptors that regulate the expression of genes involved in lipid metabolism, inflammation, and immune responses. OxLDL-derived oxidized fatty acids activate PPARγ and PPARα, leading to changes in gene expression that influence lipid metabolism and inflammation. Activation of PPARγ promotes lipid uptake and storage while also exerting anti-inflammatory effects [109,110].

However, the chronic activation of these pathways by persistent OxLDL exposure can lead to dysregulated lipid metabolism and sustained inflammatory responses. Liver X receptors (LXRs) are another group of nuclear receptors that are activated by oxysterols, oxidized derivatives of cholesterol found in OxLDL. LXR activation induces the expression of genes involved in cholesterol efflux, such as ATP-binding cassette transporters ABCA1 and ABCG1, which help to remove excess cholesterol from cells. However, excessive activation by continuous OxLDL presence can disrupt cellular lipid homeostasis [86,111].

Changes in fatty acid metabolism driven by OxLDL significantly influence the differentiation and activation of immune cells. For instance, the differentiation of macrophages into pro-inflammatory M1 or anti-inflammatory M2 phenotypes is tightly regulated by lipid metabolism [100,112,113]. In the presence of OxLDL, macrophages are more likely to adopt the M1 phenotype, characterized by high glycolytic activity, increased production of pro-inflammatory cytokines, and enhanced microbicidal functions [112,114]. The accumulation of oxidized lipids within these cells further amplifies the inflammatory response, as M1 macrophages produce large amounts of ROS and inflammatory mediators [100,106]. Conversely, the anti-inflammatory M2 phenotype, which relies more on oxidative phosphorylation and fatty acid oxidation, is less favored in an OxLDL-rich environment. M2 macrophages play roles in tissue repair and resolving inflammation, but the presence of OxLDL can impair their function, contributing to chronic inflammation and delayed resolution of inflammatory responses [100,115].

OxLDL also affects dendritic cells (DCs) by altering their lipid metabolism, which can impact their ability to process and present antigens. Enhanced fatty acid synthesis and lipid accumulation within DCs can influence their maturation and the type of T cell responses they induce [101,116]. DCs exposed to OxLDL are more likely to promote the differentiation of pro-inflammatory T cell subsets which further drive the inflammatory response [117,118].

The alterations in fatty acid metabolism induced by OxLDL have significant implications for chronic inflammatory diseases like atherosclerosis. The persistent accumulation of oxidized lipids within immune cells perpetuates the inflammatory environment, leading to the progression and instability of atherosclerotic plaques [119,120]. This environment not only supports the formation of foam cells but also contributes to the overall dysregulation of lipid metabolism within the vessel wall, exacerbating disease pathology.

### 6.4. OxLDL-Induced Mitochondrial Dysfunction

OxLDL exposure induces significant mitochondrial dysfunction in immune cells, impacting their energy production and contributing to increased generation of reactive oxygen species (ROS) [121,122]. Mitochondria, known as the powerhouse of cells, produce ATP through oxidative phosphorylation. When OxLDL disrupts mitochondrial function, it impairs energy metabolism and promotes oxidative stress, exacerbating inflammation [91].

Upon internalization of OxLDL, several deleterious effects on mitochondrial function occur. These include disruption of the electron transport chain (ETC), collapse of the mitochondrial membrane potential (MMP), increased ROS generation, and mitochondrial DNA (mtDNA) damage. The ETC, located in the inner mitochondrial membrane, is responsible for electron transfer and ATP synthesis. OxLDL disrupts the function of complexes I and III, leading to electron leakage and reduced ATP production [123,124,125]. This impairs oxidative phosphorylation, the primary pathway for ATP generation in mitochondria. The integrity of the mitochondrial membrane potential is crucial for ATP synthesis, and OxLDL-induced depolarization reduces the proton gradient required for ATP synthase activity, resulting in decreased ATP production and impaired cellular energy status [126].

OxLDL enhances ROS production within mitochondria by causing electron leakage from the ETC [127]. These ROS can further damage mitochondrial DNA, proteins, and lipids, perpetuating oxidative stress [123]. Excessive ROS activates redox-sensitive signaling pathways such as NF-κB and MAPK, promoting the expression of pro-inflammatory cytokines [128]. Mitochondrial DNA is particularly susceptible to ROS damage due to its proximity to the ETC and lack of protective histones [129]. OxLDL-induced ROS can cause mutations and deletions in mtDNA, impairing mitochondrial protein synthesis and function, further compromising respiration and energy production [130].

The mitochondrial dysfunction induced by OxLDL has several implications for immune cell function [131]. Reduced ATP production affects the energy balance within immune cells, compromising their ability to perform essential functions such as migration, phagocytosis, and cytokine production [132,133]. Energy deficiency can lead to cell death or senescence, reducing the population of functional immune cells available to respond to pathogens or damaged tissues [134,135]. Increased ROS production and mitochondrial dysfunction activate inflammatory signaling pathways. NF-κB and MAPK pathways lead to the transcription of pro-inflammatory cytokines such as TNF-α, IL-1β, and IL-6, amplifying the inflammatory response and contributing to chronic inflammation observed in diseases like atherosclerosis [128,136].

Mitochondrial dysfunction and ROS production are key activators of the NLRP3 inflammasome, a multiprotein complex involved in innate immune responses. Activation of the NLRP3 inflammasome leads to the maturation and secretion of IL-1β and IL-18, potent pro-inflammatory cytokines that further drive inflammation and tissue damage [89,137,138]. OxLDL-induced mitochondrial damage triggers autophagy, a cellular process that degrades and recycles damaged organelles [139,140]. However, chronic exposure to OxLDL can impair autophagy, leading to the accumulation of dysfunctional mitochondria. Mitophagy, the selective degradation of damaged mitochondria, is also impaired, resulting in the persistence of dysfunctional mitochondria and sustained oxidative stress [141].

In addition to the above mechanisms, OxLDL induces the opening of mitochondrial permeability transition pores (PTP). PTP opening leads to the depolarization of the mitochondrial membrane potential, further reducing ATP production [142]. This process also promotes the release of pro-apoptotic factors, increasing the rate of programmed cell death. The direct impact of OxLDL on MMP depolarization and subsequent ATP production reduction is critical. The opening of PTP and its effects on the release of pro-apoptotic factors and increased cell death are essential components of the detailed mechanisms of mitochondrial dysfunction in the presence of OxLDL [143,144].

### 6.5. Effects on Cytokine Production

OxLDL profoundly affects cytokine production by immune cells, contributing significantly to the inflammatory environment in diseases such as atherosclerosis. Cytokines are critical signaling molecules in the immune system that regulate inflammation, immune responses, and cell communication. The interaction of OxLDL with immune cells triggers intracellular signaling pathways leading to the production and secretion of various cytokines that amplify the inflammatory response [145,146].

When macrophages are exposed to OxLDL, two key pathways are activated: the NF-kB pathway and the MAPK (mitogen-activated protein kinase) pathway [147]. The NF-kB pathway, central to inflammation and immune responses, is triggered when OxLDL binds to scavenger receptors such as CD36 and LOX-1 on macrophages, leading to the activation of IKK (IκB kinase), which phosphorylates and degrades IκB, an inhibitor of NF-kB [148]. This degradation frees NF-kB to enter the nucleus and promote the transcription of pro-inflammatory genes, which results in the production of cytokines, chemokines, and other molecules that drive inflammation. At the same time, OxLDL also triggers the MAPK ERK1/2 pathway through signaling cascades involving receptor tyrosine kinases or G-protein-coupled receptors (GPCRs) [149]. Activation of ERK1/2 can occur via upstream activators often initiated by OxLDL-induced oxidative stress or receptor-mediated mechanisms. Interestingly, ERK1/2 activation is not only regulated by these external triggers but can also be enhanced by the NF-kB pathway itself. NF-kB can indirectly contribute to ERK1/2 activation by upregulating the expression of pro-inflammatory cytokines like TNF-α, which in turn can stimulate the activation of ERK1/2 through autocrine or paracrine signaling mechanisms. This creates a feedback loop where NF-kB activation promotes ERK1/2 activation and ERK1/2 enhances NF-kB’s transcriptional activity, amplifying the inflammatory response in macrophages [150,151,152]. In addition to ERK1/2, OxLDL activates other branches of the MAPK pathway, including JNK1/2 (c-Jun N-terminal kinases) and p38, which primarily respond to oxidative stress. JNK1/2 and p38 are stress kinases that help regulate the cellular response to oxidative damage and apoptosis [149]. While ERK1/2 strengthens the inflammatory response by interacting with NF-kB, JNK1/2 and p38 are more involved in cellular stress responses and apoptosis rather than directly promoting inflammation. Together, the activation of these pathways by OxLDL in macrophages highlights the dual role of OxLDL in promoting both inflammation and cellular stress, which are key factors in the development of atherosclerosis.

OxLDL can also activate the NLRP3 inflammasome in macrophages. This activation results in the cleavage and activation of caspase-1, which processes pro-IL-1β and pro-IL-18 into their mature forms. The secretion of these active cytokines further drives the inflammatory response [153]. Box 2 summarizes inflammasome biology in relation to metabolism and atherosclerosis.

Box 2 **Inflammasomes in immunometabolism and atherosclerosis.**

Inflammasomes are multiprotein complexes that play a pivotal role in innate immunity and inflammation. They are responsible for the activation of inflammatory responses by processing and secreting pro-inflammatory cytokines like interleukin-1 beta (IL-1β) and interleukin-18 (IL-18). Its key features include:

Activation: Triggered by danger signals such as oxidized low-density lipoprotein (OxLDL) and reactive oxygen species (ROS), inflammasomes activate caspase-1, leading to the maturation of IL-1β and IL-18.

 

NLRP3 Inflammasome: The most studied inflammasome, NLRP3, is activated by cellular stress and damage, including OxLDL, and contributes to chronic inflammation in diseases like atherosclerosis.

 

Role in Atherosclerosis: In foam cells, NLRP3 activation promotes the secretion of IL-1β and IL-18, fueling a feedback loop of inflammation that accelerates plaque progression and instability.

Cytokines produced in response to OxLDL play pivotal roles in the inflammatory processes underlying atherosclerosis. TNF-α is a major pro-inflammatory cytokine that promotes the recruitment of immune cells to inflammation sites. It upregulates the expression of adhesion molecules on endothelial cells, facilitating leukocyte adhesion and transmigration. TNF-α also induces other inflammatory cytokines, amplifying the inflammatory response [154]. IL-1β is a potent pro-inflammatory cytokine that contributes to inflammation by promoting immune cell activation, proliferation, and recruitment. It induces the expression of adhesion molecules and enhances the production of other cytokines, playing a significant role in the formation and progression of atherosclerotic plaques [155]. IL-6 has diverse roles in immune regulation and inflammation. It promotes the differentiation of Th17 cells, a subset of pro-inflammatory T cells, and stimulates the acute-phase response, leading to the production of C-reactive protein (CRP), an inflammatory marker associated with cardiovascular risk [156,157]. IL-18, produced through inflammasome activation, works synergistically with IL-12 to induce the differentiation of Th1 cells, which secrete interferon-gamma (IFN-γ). IFN-γ further enhances the inflammatory response and contributes to chronic inflammation [158].

**Figure 3:**
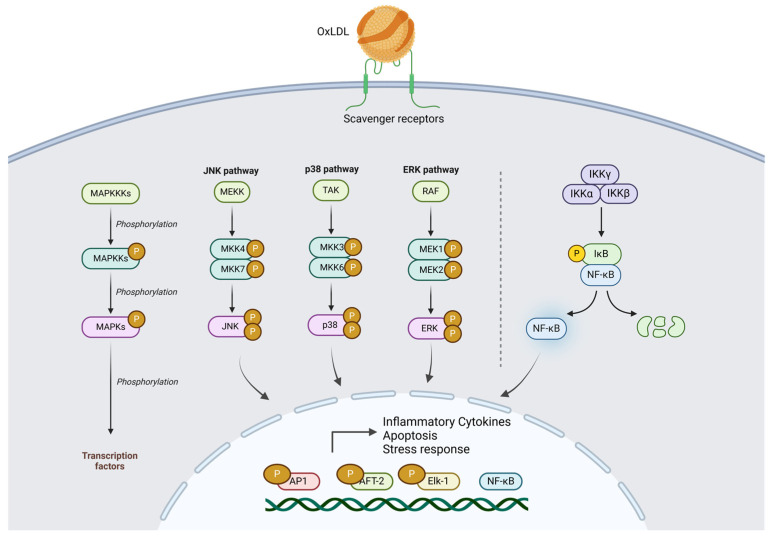
OxLDL-induced activation of MAPK (JNK, p38, ERK) and NF-κB pathways, leading to transcriptional responses that promote inflammation, apoptosis, and stress responses.

The cytokines induced by OxLDL create a feedback loop that perpetuates inflammation. For instance, TNF-α and IL-1β can enhance the production of chemokines like monocyte chemoattractant protein-1 (MCP-1), which recruits additional monocytes to the inflammation site [159,160]. These monocytes differentiate into macrophages, internalize more OxLDL, and continue the cycle of inflammation and foam cell formation. Chronic production of these cytokines can lead to systemic inflammation, affecting not only the local vascular environment but also contributing to overall inflammatory burden in the body. This systemic inflammation is linked to increased risks for metabolic syndrome, diabetes, and other chronic diseases. OxLDL-Induced Activation of MAPK and NF-κB Pathways is shown in Figure 3. 

### 6.6. Acceleration of Cellular Senescence

OxLDL significantly accelerates cellular senescence, particularly in immune cells and vascular cells, contributing to the progression of chronic inflammatory diseases like atherosclerosis [161]. Cellular senescence is a state of irreversible cell cycle arrest that occurs in response to various stressors, including oxidative stress and DNA damage. Senescent cells secrete a variety of pro-inflammatory cytokines, chemokines, and proteases, collectively known as the senescence-associated secretory phenotype (SASP), which exacerbates inflammation and tissue dysfunction [162,163]. The importance of the SASP to senescence is summarized in Box 3.

Box 3 **Senescence-associated secretory phenotype (SASP).**

The senescence-associated secretory phenotype (SASP) is a hallmark of senescent cells, which are cells that have permanently stopped dividing but remain metabolically active. The SASP involves the secretion of various pro-inflammatory molecules, including cytokines, chemokines, growth factors, and proteases. In atherosclerosis, The SASP exacerbates inflammation, recruits immune cells, and promotes tissue remodeling, which contribute to disease progression.

 

Key Components of the SASP:

Cytokines (e.g., IL-6, IL-8): Promote inflammation and immune cell activation.

Chemokines (e.g., MCP-1): Attract monocytes and other immune cells to sites of inflammation.

Proteases (e.g., MMPs): Degrade extracellular matrix components, leading to plaque instability in atherosclerosis.

Growth Factors (e.g., VEGF): Stimulate angiogenesis and contribute to vascular dysfunction.

 

Impact of SASP on Immunometabolism: the SASP promotes metabolic reprogramming in immune cells, increasing glycolytic activity and fueling chronic inflammation, which drives the progression of atherosclerosis and other chronic inflammatory diseases.

Exposure to OxLDL induces cellular senescence through several interrelated mechanisms. The uptake of OxLDL by immune cells and vascular cells generates reactive oxygen species (ROS) that cause significant oxidative damage to cellular components, including DNA [66,91]. This oxidative stress results from the imbalance between ROS production and the cell’s antioxidant defenses. ROS can cause strand breaks and base modifications in DNA, leading to genomic instability [164,165]. The persistent DNA damage activates the DNA damage response (DDR) pathway, involving key proteins such as ATM (ataxia–telangiectasia-mutated) and ATR (ATM and Rad3-related). These proteins sense DNA damage and initiate signaling cascades that result in cell cycle arrest [166,167]. The activation of DDR pathways leads to the stabilization and activation of p53, a crucial tumor suppressor protein that regulates cell cycle arrest and apoptosis. p53 activation induces the expression of p21, a cyclin-dependent kinase inhibitor that halts cell cycle progression, enforcing senescence [168,169].

Telomeres are repetitive nucleotide sequences at the ends of chromosomes that protect them from degradation and fusion. Each time a cell divides, telomeres shorten due to the end-replication problem. OxLDL-induced oxidative stress accelerates telomere shortening by increasing the rate of telomeric DNA damage [170,171]. Critically short telomeres are recognized as DNA damage, leading to the activation of DDR pathways and the induction of senescence. Telomere shortening is monitored by shelterin, a protein complex that protects telomeres. When telomeres become critically short, shelterin components recruit DDR proteins, triggering a senescence response [172,173,174]. Besides p53, the p16INK4a pathway is another crucial regulator of senescence. p16INK4a inhibits cyclin-dependent kinases 4 and 6 (CDK4/6), preventing the phosphorylation of the retinoblastoma protein (Rb). Hypophosphorylated Rb binds to E2F transcription factors, inhibiting the transcription of genes required for cell cycle progression. The inhibition of CDK4/6 by p16INK4a leads to a sustained cell cycle arrest in the G1 phase, promoting senescence. The expression of p16INK4a is upregulated in response to various stress signals, including oxidative stress and DNA damage induced by OxLDL [175,176,177,178].

Mitochondrial dysfunction induced by OxLDL also contributes to senescence. Dysfunctional mitochondria produce excess ROS, which further exacerbate oxidative damage to cellular components, including mtDNA [179,180]. This mitochondrial ROS can leak into the cytoplasm and contribute to nuclear DNA damage, perpetuating the cycle of oxidative stress and senescence. Mitochondrial dysfunction also leads to a decline in ATP production, impairing cellular energy metabolism and promoting the senescent phenotype [179,181].

Senescent cells, including those induced by OxLDL, develop a SASP characterized by the secretion of various pro-inflammatory cytokines, chemokines, growth factors, and proteases [182]. Key components of the SASP include pro-inflammatory cytokines, which create a pro-inflammatory microenvironment that attracts immune cells and contributes to chronic inflammation. Senescent cells also produce chemokines like MCP-1, which recruit monocytes and other immune cells to the site of inflammation. The recruitment of these cells further propagates the inflammatory cycle and enhances tissue damage [183,184,185]. Additionally, senescent cells secrete matrix metalloproteinases (MMPs) and other proteases that degrade the extracellular matrix, contributing to tissue remodeling and plaque instability in atherosclerosis [186]. Growth factors such as VEGF promote angiogenesis and vascular permeability, influencing disease progression [187].

The accumulation of senescent cells in the vascular wall and immune system has significant implications for the progression of atherosclerosis and other chronic inflammatory diseases. Senescent endothelial cells and vascular smooth muscle cells impair vascular function, leading to endothelial dysfunction, reduced nitric oxide availability, and increased vascular stiffness [188]. These changes promote atherosclerotic plaque formation and progression. In the context of atherosclerosis, senescent immune cells within atherosclerotic plaques perpetuate the inflammatory environment. The SASP factors secreted by these cells recruit additional immune cells, including macrophages and T cells, to the plaque, enhancing inflammation and plaque growth. The presence of senescent cells also contributes to plaque instability, as MMPs degrade the fibrous cap, increasing the risk of plaque rupture and subsequent thrombotic events such as myocardial infarction and stroke.

Mechanisms by which OxLDL may induce cellular senescence are summarized in Table 1.

## 7. Clinical and Therapeutic Implications

Given the central role of OxLDL in the pathogenesis of atherosclerosis and autoimmune diseases, several therapeutic strategies have been developed to target OxLDL and its effects. Antioxidants are agents that neutralize ROS and reduce oxidative stress, thereby preventing the oxidation of LDL. Common antioxidants include vitamins C and E, polyphenols, and specific antioxidant enzymes like superoxide dismutase (SOD) and catalase. While dietary antioxidants have shown some benefit, clinical trials have yielded mixed results regarding their efficacy in reducing cardiovascular events [189,190].

Statins are widely used lipid-lowering agents that inhibit HMG-CoA reductase, the key enzyme in cholesterol biosynthesis [191]. By lowering LDL levels, statins indirectly reduce the substrate available for oxidation, thereby decreasing OxLDL formation. Statins also have pleiotropic effects, including anti-inflammatory and antioxidant properties, which contribute to their cardiovascular benefits [192,193].

Anti-inflammatory agents have been explored as therapeutic options, including nonsteroidal anti-inflammatory drugs (NSAIDs), corticosteroids, and more targeted biologics such as monoclonal antibodies against specific cytokines (e.g., TNF-α inhibitors, IL-1β inhibitors) that can help mitigate the inflammatory response induced by OxLDL [194,195].

Inhibitors of scavenger receptors are being investigated to reduce the uptake of OxLDL by macrophages and other immune cells. By blocking these receptors, it may be possible to decrease foam cell formation and the progression of atherosclerosis.

Lifestyle modifications, including diet, exercise, and smoking cessation, play a crucial role in managing oxidative stress and reducing OxLDL levels [196,197]. Diets rich in fruits, vegetables, and whole grains, along with regular physical activity, can improve lipid profiles and reduce the risk of atherosclerosis and related cardiovascular diseases [198].

Recent advances in understanding the role of OxLDL in immunometabolism have opened new avenues for therapeutic interventions. Immunomodulatory therapies that specifically target the metabolic pathways influenced by OxLDL are being developed. These therapies aim to modulate immune cell metabolism to reduce inflammation and improve immune function. For example, drugs targeting glycolysis or fatty acid metabolism in immune cells could help mitigate the pro-inflammatory effects of OxLDL [199].

As discussed in the section on cellular senescence, senolytics (drugs that selectively induce apoptosis in senescent cells) and senomorphics (agents that modulate the SASP without eliminating senescent cells) are promising strategies to reduce the burden of senescent cells induced by OxLDL [200]. These therapies could potentially alleviate chronic inflammation and improve cardiovascular outcomes.

## 8. Future Directions and Research Gaps

### 8.1. Identification of Unresolved Questions and Key Challenges

Despite significant advances in understanding the role of OxLDL in immunometabolism and its implications for chronic diseases, several key questions and challenges remain unresolved. One major area of uncertainty is the precise molecular mechanisms by which OxLDL induces specific immune responses and contributes to metabolic dysregulation. While the involvement of scavenger receptors and downstream signaling pathways is well documented, the detailed interplay between these pathways and their cumulative effects on immune cell behavior and disease progression need further elucidation. Additionally, the heterogeneity of OxLDL particles and their differential effects on various immune cell types present a complex landscape that requires more in-depth investigation.

### 8.2. Potential Novel Targets for Therapy and Prevention

Future research should focus on identifying and validating novel therapeutic targets that can modulate the effects of OxLDL on immune and metabolic pathways. One promising area for therapeutic intervention involves targeting the key enzymes responsible for LDL oxidation, such as lipoxygenases, myeloperoxidase, and NADPH oxidase. Inhibiting these enzymes can prevent the production of OxLDL and its associated pro-inflammatory effects. Additionally, enhancing the antioxidant capacity of cells to mitigate oxidative stress represents a practical approach for reducing OxLDL levels [77].

Another novel therapeutic strategy under consideration is the use of OxLDL as an immunogen. In this approach, oxidized LDL is recognized by the immune system as an antigen, triggering immune responses. Immunotherapy or vaccination with OxLDL aims to induce immune tolerance to oxidized LDL, thereby reducing inflammatory responses. This method utilizes specific epitopes from OxLDL to elicit a protective immune response. However, this strategy carries the risk of unpredictable immune reactions. Instead of promoting tolerance, the immune system may respond erratically, leading to increased inflammation. For example, anti-OxLDL IgG antibodies may form pro-atherogenic immune complexes, which could exacerbate inflammation and potentially worsen the disease. Thus, precise regulation of the immune response is critical, as improper stimulation may aggravate rather than alleviate the condition [201].

On the other hand, anti-oxidized LDL antibodies, particularly IgM, have shown potential in binding to OxLDL, preventing its uptake by macrophages, and thereby reducing foam cell formation and the development of atherosclerotic plaques. Despite this, the long-term effects of using anti-OxLDL antibodies remain unclear. There is a possibility of unexpected side effects, such as an increased risk of autoimmune diseases or chronic inflammation, particularly in individuals with a genetic predisposition to autoimmune disorders. Furthermore, genetic and immunological variability among individuals could lead to diverse responses to this treatment, making it challenging to predict and manage immune reactions effectively across different populations [201].

In addition to these strategies, targeting scavenger receptors involved in OxLDL uptake, such as CD36, SR-A, and LOX-1, offers another potential therapeutic avenue. Inhibitors or monoclonal antibodies targeting these receptors could prevent the internalization of OxLDL by immune cells, thus reducing foam cell formation and the associated inflammatory responses [202]. Moreover, modulating downstream signaling pathways activated by OxLDL, such as NF-κB, MAPK, and the NLRP3 inflammasome, could further enhance therapeutic outcomes [89,203].

Finally, considering the role of immunometabolism in OxLDL-related diseases, targeting metabolic pathways in immune cells presents another promising approach. Drugs that modify glycolysis, fatty acid oxidation, and mitochondrial function in immune cells—such as metformin—could mitigate OxLDL-induced inflammation and improve overall metabolic health.

### 8.3. Suggestions for Future Research

Future research should adopt interdisciplinary approaches that integrate insights from immunology, metabolism, molecular biology, and clinical science to address the complexities of OxLDL and its effects. Advanced research techniques, such as single-cell RNA sequencing, proteomics, and metabolomics, can provide comprehensive profiles of immune cell responses to OxLDL at unprecedented resolution. These techniques can identify novel biomarkers and therapeutic targets by revealing the detailed molecular changes induced by OxLDL in different cell types and disease contexts.

Another important direction is the development of animal models that more accurately reflect human disease conditions. Current models often fail to capture the full spectrum of human immune and metabolic responses to OxLDL. Improved models that incorporate genetic, dietary, and environmental factors relevant to human disease could enhance the translational value of preclinical research.

Collaborative efforts between academic researchers, clinicians, and the pharmaceutical industry are essential to accelerate the translation of basic research findings into clinical applications. Large-scale, multicenter clinical trials are needed to test the efficacy and safety of new therapies targeting OxLDL and its associated pathways. These trials should include diverse populations to ensure that findings are generalizable and to identify potential differences in treatment responses based on genetic and environmental factors.

Furthermore, personalized medicine approaches should be emphasized to tailor therapies based on individual patient profiles. Genetic, metabolic, and immunological screening can help identify patients who are most likely to benefit from specific interventions targeting OxLDL. This precision medicine approach can improve treatment efficacy and reduce the risk of adverse effects.

## 9. Conclusions

OxLDL plays a pivotal role in the pathogenesis of atherosclerosis and chronic inflammatory diseases by promoting foam cell formation, inducing inflammatory responses, and contributing to plaque instability. Recognized and internalized by immune cells through scavenger receptors, OxLDL triggers metabolic reprogramming, increased glycolysis, altered fatty acid metabolism, and mitochondrial dysfunction, enhancing the inflammatory phenotype of immune cells. This leads to the secretion of pro-inflammatory cytokines and accelerates cellular senescence, perpetuating chronic inflammation. Therapeutic strategies targeting OxLDL, including antioxidants, statins, anti-inflammatory agents, and inhibitors of scavenger receptors, as well as advances in immunometabolism, offer promising avenues for treatment. However, translating these findings into clinical practice requires further research to identify novel targets, develop better animal models, and conduct large-scale clinical trials. Collaborative and personalized approaches are essential to advancing treatment strategies and improving cardiovascular outcomes.

## Figures and Tables

**Table 1:** Summary of mechanisms by which OxLDL may induce senescence.

Mechanism	Description
Oxidative Stress	ROS generation leading to DNA damage and activation of the DDR pathway, including ATM/ATR, p53, and p21, causing cell cycle arrest. Chronic oxidative stress perpetuates the stress response, contributing to senescence.
Genomic Instability	Accelerated by ROS, leading to DNA damage and genomic instability, activating the p16INK4a pathway and inhibition of CDK4/6 and resulting in sustained cell cycle arrest and senescence. Telomere shortening is also a key factor in this process.
Mitochondrial Dysfunction	Disruption of the electron transport chain leading to reduced ATP production, increased ROS, and activation of the NLRP3 inflammasome. Mitochondrial dysfunction drives chronic inflammation (inflammaging) and cellular senescence.
Senescence-Associated Secretory Phenotype (SASP)	Secretion of pro-inflammatory cytokines, chemokines, growth factors, and proteases, which exacerbate inflammation and tissue remodeling. SASP also impacts the tissue microenvironment, potentially promoting tumorigenesis.
